# Human Health Risk Assessment to the Consumption of Medicinal Plants with Melliferous Potential from the Romanian South-Eastern Region

**DOI:** 10.3390/toxics11060520

**Published:** 2023-06-09

**Authors:** Lucica Barbeș, Alina Bărbulescu, Cristian Ştefan Dumitriu

**Affiliations:** 1 Department of Chemistry and Chemical Engineering, “Ovidius” University of Constanța, 124 Mamaia Bd., 900112 Constanta, Romania; lucille.barbes2020@gmail.com; 2 Doctoral School of Biotechnical Systems Engineering, Politehnica University of Bucharest, 313 Splaiul Independentei, 060042 Bucharest, Romania; 3 Department of Civil Engineering, Transilvania University of Brașov, 5 Turnului Str., 900152 Brasov, Romania; 4 Faculty of Mechanical and Robotic Engineering in Construction, Technical University of Civil Engineering of Bucharest, 124 Lacul Tei Av., 020396 Bucharest, Romania

**Keywords:** melliferous plants, pollution indicators, statistical analysis

## Abstract

This study presents the impact on human health by consuming medicinal herbs with high melliferous potential (HMPs) from botanical areas with different pollution levels. First, the bioaccumulation of the plants’ parts has been determined. The study assessed the potential health risks associated with the ingestion of various mineral species (macroelements—K, Ca, Mg, Na; microelements—Fe, Mn, Cu, Zn, and one trace element Cd) from three types of HMPs (*Sambucus nigra* (*SnL*), *Hypericum perforatum* (*Hp*), and *Tilia tomentosa* (*Tt*)). The average concentrations of these elements were not similar even in the same type of HMPs. Nevertheless, all samples contained detectable levels of the studied elements. The average concentrations of the studied elements were very low (significantly lower than the legal limit set by the WHO). The study’s findings indicated that the potential health risks associated with ingesting the elements in HMPs were within acceptable limits for children and adults. The hazard quotient (HQ) for Fe, Mn, Cu, Zn, and Cd and the hazard index (HI) for the minerals from HMPs were significantly lower than the acceptable limit (HQ and HI = 1). Similarly, the carcinogenic risk for chemical substances (${\mathrm{Risk}}_{\mathrm{ccs}}$) were lower than or close to the acceptable limit (1 × 10^−4^).

## 1. Introduction

It was shown that honey and derived products borrow the origin plant properties, containing elements that act as antibiotics [1,2]. Medicinal plants, including those with melliferous potential, are frequently used in tea preparation. Therefore, the chemical elements (some with a potentially toxic effect) are transferred from the plants to the tea infusions and further reach the human body after consumption. Medicinal plants are species that accumulate various active principles in some of their parts so that their therapeutic properties are used to treat different diseases. They have a long history of use in different forms of natural therapy [3,4]. In the USA, 25% of drugs are plant drugs, whereas the percentage is much higher in India or China (80% of total medicines). In the meantime, the biological activity of only 6% of the known species has been investigated [5], among which smaller attention has been paid to the plants with melliferous potential [6,7,8,9].

Medicinal plants used for therapeutic, culinary, or cosmetic purposes are subject to primary and secondary processing, from which they are utilized in different forms: in their natural state (fragments, powders, etc.), infusions, decoction, maceration or phytotherapeutic products for internal use or externally in the form of extracts, tinctures, syrups, aromatic oils, derived bee products, etc. [10,11].

Plants with honeydew potential are plants whose pollen is transformed mainly into honey by bees. Most honey plants can fulfill a double role, being also medicinal plants. They can be used as they are or processed in other derivatives in the food and cosmetics industries [12,13]. The area of search for pollen by fodder bees is estimated at up to 28 km^2^, covering a plant diversity that can influence the dimension and quality of the melliferous products.

Global biodiversity is threatened by intensive agriculture, land use, and climate change [7,8,14]. Environmental pollution (which increases the toxic metallic content in the soil), together with the natural plant affinity to some elements, can lead to the plants’ contamination [15,16,17,18], especially by pesticides and metallic elements [19,20]. For example, the carbonate dissolution and the pH variation in the rhizosphere soil may augment the mobility of Cu and Zn, followed by the metals’ (Pb, Mn, and Cu) accumulation in the leaves [21,22]. Additionally, a pH decrease is associated with an augmentation of Cd mobility [23,24]. Microelements, macroelements, and trace elements released from various sources into the atmosphere can be transported, deposited, and absorbed by the plants, leading to their contamination [25,26]. Plants’ uptake of toxic substances from soil and their subsequent accumulation along the food chain is a potential threat to human health [27,28]. It was shown that the accumulation of Cd, Ni, Pb, As, Hg, etc., in the soil where the melliferous plants grow, could lead to the contamination of the honey and propolis [1,29]. To argue this idea, some researchers [30,31,32,33] evaluated the accumulation of heavy metals in honeybees in different regions of the world as an effect of the pollution of the bees’ habitats. Others showed that exposure to some metals through the natural consumption of medicinal plants or derived products (such as aqueous or alcoholic extracts [34,35,36,37,38], bee products [2,39,40,41], cosmetics [42,43], or even homeopathic medicines [27] negatively impact the human health. Luo et al. [27] analyzed the risk exposure to some medicinal plants used as food ingredients and herb supplements as herb capsules. The authors considered the standard amount contained in a 500 mg/capsule and the daily intake of two capsules, leading to a total ingestion of 1 g/day. This value was also used to calculate the estimated daily intake (EDI, mg/kg/day) of different metals and hazard quotient (HQ) for the non-cancer risk evaluation. The weight of an average adult was considered to be 60 kg, according to the FAO/WHO [44] documents. Additionally, the authors suggested different solutions for managing heavy metal levels in herbal medicines. They considered it crucial to establish a consistent monitoring and surveillance plan to regulate external contamination of herbal medicines throughout the supply chain, from cultivation to consumption.

Still, very few studies evaluated the health risks of consuming medicinal plants with melliferous potential [6,9,29,33,45]. In such investigations, specific indicators were computed [46,47,48,49,50,51,52] and compared with some critical levels to indicate the level of danger. The results have shown that medicinal plants must be carefully used, considering their possible adverse effects on the human organism [53,54,55,56,57,58].

The medicinal plants harvested directly by consumers from the spontaneous flora without testing them adequately can have long-term health consequences. That is why it is considered necessary to assess the tolerable daily intake (TdI), provisional tolerable daily maximum intake (PtdmI), and provisional tolerable weekly intake (PtwI) for all identified chemical elements because some are potentially toxic [35]. Daily consumption of plant-derived products (aqueous extracts) can cause significant health risks when the intake of chemical elements with a potentially toxic effect exceeds specific values established for the dietary reference intakes (DrI). For assessing the highest safe daily amount of nutrient intake, the tolerable upper intake level (TuiL) (often regulated by each state’s authorities, is taken into account) or the values of some toxicological indices adopted by various specialist agencies are taken into account. However, the permissible limits for toxicological parameters are not established for all elements. Therefore, the results of different studies are very important to evaluate the health risks. Some values of interest for our study, indicating the maximum intake of specific elements per kg of body, day or week, are presented in [35]: Cd (7 μg/kg/week), Cu (40 μg/kg/day), Fe (700 μg/kg/day) [59], Mn (140 μg/kg/day) [60], and Zn (0.3 mg/kg/day). The following values were considered for the macroelements: 2.3 g (Na) [61,62], 6 g/day (K) [63], 2.5 g (Ca) [64], and 0.25 g (Mg) [65]. The proposed limit of monthly consumption of Cd is 25 μg/kg, although no intake level is considered safe for cadmium.

Another possibility to evaluate the health risk due to food consumption is based on the following indices [42,50,51,66]: the estimated daily intake (EDI) or exposure assessment, non-carcinogenic risk assessment (the Hazard Quotient Index (HQ), and the Hazard Index (HI)), and carcinogenic risk for the chemical substances (Risk_ccs_). The U.S. Environmental Protection Agency (EPA) replaced TdI with the Reference Dose (RfD). The RfD is adopted to explain if the consumption of herbal derivative products is entirely safe or acceptable.

It should be noted that metallic elements have an essential role in the multitude of reactions that lead to the formation of the constituents of the plant rich in active principles and, therefore, are responsible for the most part for their curative but also toxic properties.

Given the impressive development of beekeeping in Romania over the last two decades, this study assesses the contamination levels and the risk for human health presented by metallic species, especially in products derived from medicinal plants (teas) with honey-bearing potential from spontaneous species harvested from the Northern Dobrogea Plateau, Romania.

In the described context, the study aims to investigate the content of some macro-, micro-, and trace elements in honeydew plants from the south-eastern region of Romania through FAAS and the statistical analysis of the concentration variations in these minerals in the studied plant species. Additionally, the article evaluates the risk to human health associated with the consumption of infusions from medicinal plants with honey-like potential, using weighted averages of the concentrations of the analyzed chemical elements and their comparison with the specific food safety standards and limits [44].

The original aspects of this interdisciplinary study consist of the following:

(1) The analysis of the metal content in spontaneous species with honey and medicinal potential from the studied area in 2019 (some of which have been a little bit studied until now) for the Dobrogea region, an important region with melliferous potential in Romania;
(2) The determination of some indicators in the evaluation of the toxicity of metal species from three species of honeydew plants harvested from the North Dobrogea Plateau;
(3) The computation of the weighted averages of the daily intake of macroelements and microelements (daily mineral intake—expressed in mg/kg/day) for an adult from infusions of medicinal plants harvested during the flowering period of 2019 for the analyzed species (May–June: *SnL*, May–September: *Hp*, and June–July: *Tt,* respectively). The obtained values were compared with the weighted averages of the maximum tolerable daily ingested mineral content (TuiL = tolerable upper intake level, mg/day) by an adult for the same analyzed mineral elements. This evaluation method has not been applied until now.

## 2. Materials and Methods

### 2.1. Study Area

The south-eastern Romanian region presents a great biological diversity, including honeydew flora as an important part of Romania’s honeydew heritage, which needs to be more exploited.

For a correct systematization and appropriate classification of plants with melliferous potential, some selection criteria are established in the literature [67] as follows:

(1) The botanical origin. For example, lime, which is part of the order Malvares, the family *Tiliaceae*, and the genus *Tilia* with the species *Tilia cordata*, *Tilia tomentosa*, and *Tilia platyphyllos*);
(2) The biological and economic classification: trees and shrubs, spontaneous herbaceous plants (like those in forests, hayfields, and meadows, or those that appear spontaneously in crops, etc.), cultivated plants (including aromatic medicinal plants, technical plants, fodder plants, leguminous plants, and, specifically, melliferous plants of the type of fruit trees);
(3) The nature of the food made available to the bees by the plants: nectariferous (provides only nectar); polliniferous (from which bees collect only pollen); nectar polliniferous (for example, linden, acacia, sunflower); manna producing (for example, fir or spruce). The flowers of these plants react differently to specific pollinators (especially bees), providing them, in different flowering periods, colors, scents, shapes, and rewards (pollen or nectar).

Forest ecosystems have a significant potential for food and medicinal resources [5]. By blooming from February/March to the end of October, these plants provide bee families with pollen and nectar throughout the active beekeeping season. According to the Romanian Beekeeping Law [68], all the plants’ bees use to search for nectar, honeydew, or pollen to form a melliferous base.

**Figure 1:**
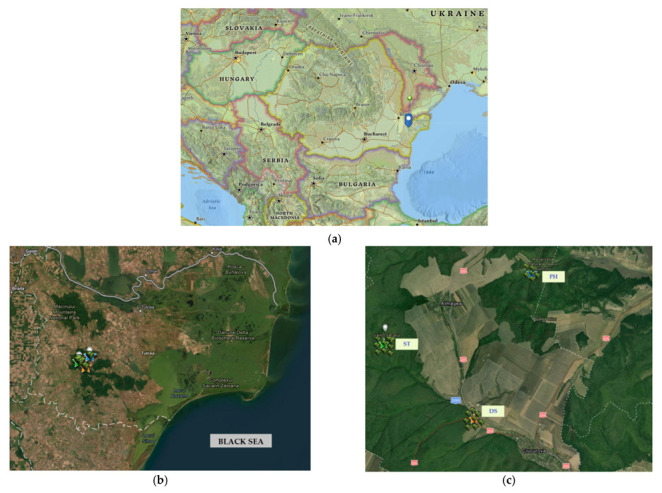
(**a**) East Europe map; (**b**) Map of the Tulcea county (România) where the experimental sites are situated; (**c**) Izvorul Cerbului (DS), Dealul Bujorului (PH), Vârfu Secaru (ST).

The region from where the plants have been collected was situated in Romania, in the Northern part of the Dobrogea Plateau, in the vicinity of the Ciucurova forest (Figure 1).

The vegetation consisted mainly of forests, where the *Tilia* spp. were found at altitudes from 70 to 400 m, occupying 45% of the surface. Among the frequently encountered medicinal plants, there were various species, including Adoxaceae and Hypericum.

Two sampling areas were the natural reserves Vârful Secaru, Secaru Hill Top (ST) (44°57′14′′ N; 28°24′19′′ E) and Dealul Bujorului, Peony Hill (PH) (44°57′35′′ N; 28°29′00′′ E) on the Babadag Plateau. These reserves had areas of 34.5 ha and 50.8 ha, respectively [6]. The third location was the Ciucurova forest, near the Izvorul Cerbului, Deer Spring (DS) camping (44°55′33.5′′ N; 28°26′54.1′′ E).

The soil was of the chernozem type, with an acidity of around 7. A continental-type climate with maritime influences, hot summers, cold springs, and low quantities of precipitation was characteristic of the region [6]. The lands’ deteriorations were caused by natural hazards (especially erosion), traffic pollution, and the waste chaotically deposited in the touristic zones.

### 2.2. Experimental Study

Samples of *Sambucus nigra* (*SnL*), *Hypericum perforatum* (*Hp*), and *Tilia tomentosa* (*Tt*) and soils were collected in May–August 2019 from DS, PH, and ST zones by following the procedure. Ten inflorescence samples (for each plant) were collected. Samples of *Tt* were collected from 30 trees (15 trees per site) aged 20–30 years, 25–35 m tall, with big branches, thick grey-brown barks, and small leaves, without mechanical or biological damages. The trees were located approximately 15 m from each other. At least 300 g of fresh flowers were collected from each tree, at the height of 2.5–3 m above the ground, by evenly including all directions and cutting off at least four branches around the crown periphery of each tree. All samples were washed with bidistilled water. The samples of plant tissues were dried in an oven at 70 °C for 24 h. A mixer grinder was used to powder the samples while preventing overheating [7].

The analysis of metallic species (macroelements, microelements, and trace elements) was frequently carried out by analytical methods (AAS—atomic absorption spectroscopy; AES—atomic emission spectrometry; and/or ICP—inductively coupled plasma spectroscopy) [45,69,70].

The analysis of the concentrations of elements in samples was performed by FAAS [16,71] using a ContrAA^®^ 700 spectrometer, with a high-resolution double-scale monochromator optical system, its spectral resolution in the interval 0.002–200 nm, and the wavelength between 185 and 900 nm [6,7]. A quantity of 0.5 g of powder of the plants’ dry flowers was mineralized with 5 mL nitric acid and 40 mL deionized water to 120 °C for 130 min. The clear solutions were filtered and stored in 50 mL volumetric bottles with distilled deionized water. The content of elements in the plants’ parts was determined by employing the calibration curve method, as presented in [16]. The experiments were performed in triplicate, according to NIST SRM 2709, 2710, and 2711 [7,16]. The recovery average for the analyzed elements (Ca, Cd, Cu, Fe, K, Na, Mg, Mn, and Zn) varied between 76% and 92%, with standard deviations (SD) between 0.001 and 0.0036.

The tea infusion process was carried out as follows. Two grams of dried inflorescence plants were put into a 250 mL Erlenmeyer flask, and 200 mL of boiling water (ultrapure demineralized) was added. The tea infusion was mixed using a glass rod to ensure adequate wetting and then covered for 10–15 min. After the infusion process, the obtained solutions were decanted and cooled to room temperature until analysis was performed using FAAS-based methodology. All analyses of the tea infusion samples were conducted in triplicate. The recovery yields, detection limits, and SD for triplicate measurements of the elements were 91–98%, less than 3 × 10^−11^, and less than 5%, respectively.

### 2.3. Health Risk Assessment of Metal Accumulation by Herbs Consumption

The risk assessment of herbal plants involved four steps: hazard identification, exposure assessment, toxicity assessment, and risk characterization [72]. Thus, the risk depended on toxicity and exposure.

The toxicological indexes used in the present research are given in Equations (1)–(10).

The reference dose (RfD) (mg/kg/day) (Table 1) was defined by:

(1) RfD= NOAEL/(UF×MF),

where

- NOAEL—the no-observed-adverse-effect level (defined as the dose experimentally determined at which no biologically or statistically significant evidence of the toxic effect was detected);
- UF and MF were the uncertainty and modifying factors, respectively [73];
- MF was a supplementary uncertainty factor, taking values between 0 and 10, depending upon different factors (tested species, data bases quality, etc). By default, MF = 1 [73].

**Table 1:** The oral reference dose (mg/kg/day) by ingestion exposure [42,74].

Element	Adult	Product	References
Cd	0.001	medicinal herb/infusion tea	[27]
	0.000001		[48]
Cr	0.003	medicinal herb	[27]
	0.14	honey	[75]
Cu	0.04	medicinal herb	[27]
	0.5	honey	[75]
Fe	0.7	medicinal herb	[27]
Mn	0.14	medicinal herb	[27]
	1.5	honey	[75]
Ni	0.02	medicinal herb	[27]
Zn	0.3	medicinal herb	[27]
	1	honey	[75]

The potential dose (PD) (in mg/kg of body weight per day) was the quantity of the ingested contaminant (by mouth), computed by [76]:

(2) $$\mathrm{PD}={\;C}_{\mathrm{ce}}\times \;\mathrm{IR},$$

where $C_{\mathrm{ce}}$ was the concentration of the element in a plant (mg/kg dry weight), and IR was the ingestion rate of the chemical element (g/person-day for dried plant) or the average infusion’s consumption rate (mL/person-day for plant infusions).

The chronic daily intake of a chemical element (CDI) (mg/kg of body weight per day) was computed using (3) or (4):

(3) $$\mathrm{CDI}={\;C}_{\mathrm{ce}}\times \;\mathrm{IR}/\mathrm{BW},$$

(4) $$\mathrm{CDI}={\;C}_{\mathrm{ce}}\times \;\mathrm{IR}\times \mathrm{ED}\times \mathrm{EF}\times \mathrm{TR}/\left(\mathrm{BW}\times \mathrm{AT}\times 1000\right),$$

where

- ED was exposure duration in years (for example, 20 years for a shorter-than-lifetime period and 70 years for an average lifetime, respectively);
- EF was the exposure frequency (days/year, i.e., 90 days, or events/day, i.e., 2–3 events);
- TR was the transfer rate of metals from dried medicinal plants to preparations (*w*/*w* %);
- BW was the average body weight of the general population in Romania (70 kg for adults and 15 kg for children);
- AT was the average exposure time to herbal plant or tea infusion consumption (365 days/year × 70 years = 25,550 days for adults and 365 days/year × 10 years = 3650 days for children) [34,77];
- A total of 1000 was the conversion factor for the mass unit (from mg to kg).

The hazard quotient (HQ) (dimensionless) was the ratio between the chronic daily intake and the reference dose:

(5) HQ=CDI/RfD,

HQ evaluated the potential risk of noncarcinogenic chronic damage to human health. When HQ > 1, there was a hazard a potential.

The hazard index (HI) (dimensionless) was the sum of the hazard quotients for exposure to many chemical elements:

(6) $$\mathrm{HI}=\sum_{i=1}^{n}\mathrm{HQ}$$

where *i* was the number of such elements.

A value HI < 1 indicated no significant noncarcinogenic risk, whereas HI > 1 proved the occurrence of a noncarcinogenic health risk. The values of HI above 10 showed a significant chronic health impact [46].

The carcinogenic risk for a chemical substance or its Lifetime Cancer Risk ${\mathrm{Risk}}_{\mathrm{ccs}}$ (dimensionless) was defined by:

(7) $${\mathrm{Risk}}_{\mathrm{ccs}}=\mathrm{CDI}\times \mathrm{SFC},$$

where CDI was the chronic daily intake of a chemical element (mg/kg/day), and SFC was oral slope factor carcinogenic (mg/kg/day) (Table 2).

The acceptable standard cancer risk was between 10^−6^ to 10^−4^ [81]. If the carcinogenic risk value exceeded 10^−4^, it was considered an unacceptable level of carcinogenic risk over an individual’s lifetime [78]. The cumulative carcinogenic risk for many chemical substances ${\;\mathrm{CRisk}}_{\mathrm{ccs}}\;$(dimensionless) represented the sum of the risks for all the carcinogenic chemical substances:

(8) $${\;\mathrm{CRisk}}_{\mathrm{ccs}}=\sum_{i=1}^{n}{\left({\mathrm{Risk}}_{\mathrm{ccs}}\right)}_{i}$$

where *n* was the number of the considered elements, and ${\left({\mathrm{Risk}}_{\mathrm{ccs}}\right)}_{i}$ was the carcinogenic risk of the *i*-th element.

If the CRisk_ccs_ < 1, no carcinogenic effect was expected. If CRisk_ccs_ > 1, some protective measures must have been taken because there was a potential health risk. If people were exposed to more pollutants simultaneously, the effects were the sum of individual effects or more. The cumulative risk of all elements was used to estimate the potential health effects expected as a consequence of exposure or ingestion and to evaluate the adverse effects of herbal extract (tea or infusions) consumption.

For the calculation of the weighted averages of the daily intake of macroelements and microelements (provisional daily mineral intake PDMI—expressed in mg/kg/day) from infusions of medicinal plants for an adult with an average body weight of 70 kg, the following relationships were used:

(9) $$C_{\mathrm{mp}}=\overset{n}{\underset{i=1}{\sum }}\left(C_{\mathrm{ce}}\times \mathrm{TR}\right),$$

(10) $$\mathrm{PDMI}={\;C}_{\mathrm{mp}}\times \;\mathrm{IR}\times \mathrm{EF}/\left(\mathrm{BW}\times \mathrm{AT}\times 1000\right),$$

where

- C_ce_ was the average concentration of the chemical element in a plant (mg/kg dry weight);
- TR was the transfer rate of metals from dried medicinal plants to infusion (*w*/*w* %);
- C_mp_ was the weighted average concentration of the mineral elements in a plant infusion (mg/L for plant infusion);
- IR was the average infusions consumption rate (mL/person-day for plant infusions);
- EF was exposure frequency (days/year, i.e., 14 days/year, or events/day, i.e., 2–3 events);
- BW was the average body weight of the general population in Romania (70 kg for adults and 15 kg for children);
- AT was the average exposure time to herbal plant or tea infusion consumption (365 days/year)
- A total of 1000 was the conversion factor (from mL to L).

The transfer rate from the plant into the tea is computed by [36]:

*T* = *c × v* × 100/(*w* × *m*), (11)

where *T* was the transition rate in %;

- *w* was the content of the element in the plant (μg/g);
- *c* was the content of the element in the brew (μg/L);
- *v* was the volume of preparing the infusion (200 mL);
- *m* was the plant quantity for preparing the tea (2 g).

In this study, it was considered that the consumption of one adult was three cups of tea per day and one cup of tea per day for a child (prepared as explained in Section 2.2).

The studies analyzing tea consumption did not consider the total or partial transfer of minerals from the tea to the human body. It was supposed that the entire quantity of the mineral was absorbed by the organism in some conditions, whereas only a part was absorbed in other conditions. Therefore, the computation was performed in the worst case, which was when all the elements were entirely absorbed [36,82,83,84].

## 3. Results and Discussion

### 3.1. Analysis of the Mineral Accumulation in Herbs

The results of the mineral composition analysis for the inflorescences of the three biological species studied from May to August 2019 are presented in Table 3.

The minimum average concentrations of the macroelements varied in the order Ca > K > Mg> Na, and, for the maximum values, in the order K > Mg > Ca > Na, as follows: Ca (min. 1.35 mg/kg d.w.—*Hp* at DS; max. 6.85 mg/kg d.w.—*SnL* at ST), K (min. 0.55 mg/kg d.w.—*Hp at* DS, max. 48.67 mg/kg d.w.—*SnL* at ST), Mg (min. 0.12 mg/kg d.w.—*Tt* at DS; max. 15.82 mg/kg d.w.—*SnL* at ST), and Na (min. 0.03 mg/kg d.w.—*SnL* at ST, max. 0.15 mg/kg d.w.—*Hp* at ST).

For microelements, both minimum and maximum average concentrations varied in the order Fe > Mn > Zn > Cu, as follows: Fe (min. 61.39 mg/kg d.w.—*Tt* at PH, max. 193.6 mg/kg d.w.—*Hp* at DS), Mn (min. 16.67 mg/kg d.w.—*Tt* at PH, max. 146.48 mg/kg d.w—*Hp* at ST), Zn (min. 12.46 mg/kg d.w.—*Tt* at ST, max. 53.13 mg/kg d.w.—*Hp* at DS), and Cu (min. 0.41 mg/kg d.w.—*SnL* at PH, max. 24.61 mg/kg d.w.—*Hp* at DS).

Sources of macronutrients, microelements, and trace metals in herbs include the soil base [6], atmospheric quality [17], manufacturing process, and storage [62]. Plant mineral content is influenced by factors such as their origin, ripening stage, climatic conditions, and soil pollution [85]. However, one of the most significant factors determining mineral content is the genetic composition of the plants. In addition, concentrations of elements in plants can change during vegetation season. Moreover, there are circadian variations in element concentrations in plants and rhizosphere soil. These changes can be significant during the day and quite different for the plant species, even though they grow in the same site. For the small quantities of potassium in the plants (especially in Hp and Tt), the main causes are aeration deficit, compaction, water absence (Dobrogea is the most-arid region in Romania, with long periods without or deficient precipitation—sometimes between 4 and 6 months), soil temperature, and poor drainage.

Comparisons of the mineral content in the plants in our study are provided in the following. The mineral content analyzed for the inflorescences of SnL were different from those reported by Młynarczyk et al. [57] in fruits and flowers. The average macroelements contents presented in this study were 5–10 times lower than those reported in [57]. The situation was the opposite for microelements. The content of them we determined was approximately 2–3 times higher than those from [57]. Furthermore, the values of average concentrations determined for the element Cd in the SnL inflorescences varied between 0.001 mg/kg d.w. (PH) and 0.1 mg/kg d.w. (ST), which were comparable to the values found in SnL fruits (0.039–0.053 mg/kg d.w.) in [57]. From the analysis of the mineral content of the Hp inflorescences, it resulted that the concentrations determined for the macroelements were very low (K, Mg, Na < 1 mg/kg d.w., respectively, Ca < 3.5 mg/kg d.w. in the three analyzed sites) compared to those recorded by Helmja et al. [86] for the aerial part of the plant and in different aqueous or alcoholic extracts. The same authors showed that a larger number of metals was found in water-based extracts (with K being around 70% and Zn, Mn, Mg, Co, and Cr between 10–25%), whereas the metal concentrations in ethanol-based extracts were approximately 10–20 times lower. In addition to the mineral elements identified in this study, metals such as Na, Fe, and Ni were also identified in the Hp plant. The research data showed that dried powdered leaves and flowers of Hp contained higher levels of Cu, Co, and Pb compared to commercial tinctures of the plant.

Gomez et al. [87] found Cd concentrations between 0.05 and 0.26 mg/kg d.w. in the Hp plant, comparable to those we determined in the plant inflorescences, whose average values were between 0.12 and 0.92 mg/kg d.w.

The average concentrations of the microelements determined in the inflorescences of Tt species were: 6.07–20.56 mg/kg d.w. Cu, 16.67–21.32 mg/kg d.w. Mn, and 12.46–20.56 mg/kg d.w. Zn, values comparable to those presented by Petrova and Petkova [88] for Cu (4.5–7.1 mg/kg d.w.) and Zn (12.46–20.56 mg/kg d.w.), respectively, and values approximately five times lower for Mn (60–115 mg/kg d.w.). The concentrations of Cu, Mn, and Zn indicated in [86] were determined in the leaves of the same botanical species. The order of mineral elements bioaccumulation for the Tt species was similar to the order we found: Mn > Zn > Cu > Cd. For Cd, Petrova and Petkova [88] determined values between 0.13 and 0.17. The average values found in this study were between 0.08 and 4.67 mg/kg d.w., corresponding to the DS site (considered the most polluted of those we evaluated).

The levels of macroelements, microelements, and trace elements present in plants are influenced by their root’s absorption capacity or by the accumulation of dry and wet deposits on external organs such as leaves or tree bark. The mobility of metal species is mainly affected by the pH values of the soil, considering that the solubility of exchange elements is higher in slightly acidic environments. Some studies have revealed that several chemical elements can be present in high concentrations in both soil and vegetation, even if they are essential nutrients, such as Cu or Zn. For instance, Zn, Ni, and Cd have high mobility and bioavailability and are rapidly absorbed by plants. In contrast, Cu, Cd, and Pb are intermediate-mobility elements; Cd and Pb are non-essential and highly toxic [6,7,14,16,17]. Precise identification of the abundance of trace elements in the environment necessitates collecting and processing a higher number of samples.

### 3.2. Analysis of the Transfer of Mineral Elements from Herbs in the Tea Infusions

Table 4 shows the maximum values of the transfer of mineral elements from medicinal plants in tea infusions for 10–15 min. The maximum values obtained for the transfer rate of mineral content in infusion tea varied between 5 and 90%, as follows: K (50–70%), Ca (14–46%), Mg (16–40%), Na (26–65%), Fe (8–35%), Mn (6–90%), Cu (18–80%), Zn (25–80%), and Cd (5–35%).

The concentrations of macroelements, microelements, and trace metals in the analyzed MPHs and the plant infusion samples were comparable to previous results for the same or similar medicinal plants [19,46,51,77] or tea samples [34,36,38,48] and other herbal-derivative products [28,33,35,52]. The concentrations of Cd, Cu, Mn, and Zn in the analyzed infusions of *SnL*, *Hp*, and *Tt* plants were comparable to previous results for tea samples [34,48] and other tea products consumed in different continents (i.e., Africa, Europe, and Asia) [52,89,90]. The Cd, Cu, Mn, and Zn concentrations were between 0.008–0.126, 5.9–43.3, 228–2040, and 19.5–73.2 mg/kg, respectively [89]. Mineral concentrations for all samples measured in [90] were lower than China’s standards for metal limit for tea, 30 and 1.0 µg/g for Cu and Cd, respectively. Therefore, tea drinkers were not at risk after consuming these herbal preparations.

### 3.3. Results of Health Risk Assessment of Metal Accumulation by Herbs Consumption

Table 5 shows the calculated values for the average chronic daily intake of mineral elements (CDI, mg/kg body weight per day) for a child with an average body mass of approximately 15 kg and an adult with an average weight of 70 kg.

Table 6 and Table 7 show the calculated values for the hazard quotient (HQ) of microelements and trace elements, and, respectively, the hazard index (HI), both for a child and an adult, respecting the body mass considered for each category.

The HQ minimum values for microelements and trace elements were 0.1 × 10^−4^ (Cd—*SnL* at PH) for an adult and 5.3 × 10^−1^ (Mn—*Hp* at ST) for a child. The HI values were generally less than 1—between 4.5 × 10^−2^ (*SnL* at PH) and 8.49 × 10^−1^ (*Hp* at ST)—except for *Hp* in DS (1.11) for a child. For an adult, the values were in the interval 1.5 × 10^−2^ (*SnL* at PH)—2.4 × 10^−1^ (*Hp* at DS).

Table 8 presents the cumulative carcinogenic risk of the studied chemical elements. All values are between 8.66 × 10^−6^ (*SnL* at PH) for an adult and 7.57 × 10^−3^ (*Hp* at DS) for a child. All are under one, indicating that there is no significant carcinogenic risk.

The results for the daily intake of microelements (provisional daily mineral intake PDMI—expressed in mg/kg day) from infusions of medicinal plants, for an adult with an average body weight of 70 kg, based on the weighted averages of the concentrations of transferred elements, are given in Table 9. The weighted averages were computed as follows. Considering *n* elements, with the concentrations$\;c_{i}$ ($i=\bar{1,n}$), first, the percentage of each element in the mixture is computed by $w_{i}=c_{i}/\overset{n}{\underset{k=1}{\sum }}c_{k}$ ($i=\bar{1,n}$), then the weighted average concentration will be $\overset{n}{\underset{k=1}{\sum }}c_{k}w_{k}$.

The RTuiL values are indicated in the same table. They represent the percentage of element ingestion from the daily consumption of tea-based infusions (from the analyzed medicinal plants) with respect to the TuiL (mg/day) of microelements that can come from daily food consumption.

The human risk assessment indices computed for the different herbal plants did not exceed the daily dose established by WHO [91]. There were metallic elements, such as As [27], Pb [27], Cr [46,52], and Cd [46], whose values exceeded the allowed level (e.g., Cr − HQ > 1, and HI > 1.4, respectively, in the herbal preparation with *Urtica simensis, Trigonella Foenum-graceeum, Calpurnia aurea* [52].

Given that some herbs are used as supplements or teas and as food ingredients, it is important to consider this additional source of potential health risks. Although the analyzed medicinal plants with melliferous potential (*SnL*, Hp, and *Tt*) and the infusions from these plants that were evaluated, in particular, for the mineral content of cadmium, Cu, Fe, Mn, and Zn, did not exceed the proposed limits by WHO, there are no values established in Romanian legislation for these chemical elements, which can be considered safe for consumption because even small doses can cause health problems. Therefore, their mere presence in various food/medical preparations, or as such, can be considered a risk to human health.

## 4. Conclusions

Determining the content of chemical elements in medicinal plants with melliferous potential and beyond is important for several reasons: to verify the degree of purity of the plants harvested from the forest area and its surroundings through the qualitative and quantitative analysis of all potentially toxic/carcinogenic elements and implicitly to establish the traceability of these elements from the plant to the tea infusion and human organism. This analysis and risk assessments help increase food safety, respectively, and the effectiveness of herbal preparations used for medical purposes. The findings show that the plants exhibited various accumulation levels of microelements and trace elements. Almost all of the elements investigated (including K, Ca, Mg, Na, Fe, Mn, Cu, Zn, and Cd) were found to accumulate in the plants, with the highest accumulation observed in the case of *Hp* (whose stems can reach, in general, up to 30 cm high from the ground, presenting the highest risk of contamination with the elements found/analyzed), respectively, *Tt*, whose bioaccumulation of metal species originating from the soil and the water circuit in nature can be multiplied by the adsorption of elements from the atmosphere, also taking into account the fact that the aridity of the Northern Dobrogea area has increased in recent years.

The following maximum HQ for each chemical element was found: Fe—child 6.4 × 10^−2^ and adult 1.4 × 10^−2^—*Hp* (DS); Mn—child 5.3 × 10^−1^—*Hp* (ST) and adult 3.2 × 10^−1^—*Hp* (DS); Cu—child 3.2 × 10^−1^ and adult 6.9 × 10^−2^—*Hp* (DS); Zn—child 9.3 × 10^−2^ and adult 2.0 × 10^−2^—*Hp* (DS); and Cd—child 4.0 × 10^−1^ and adult 8.6 × 10^−2^—*Tt* (DS). All HI values were less than 1, but *Hp* in DS was 1.11 × 10^0^ for a child, whereas it was less than 2.4 × 10^−1^ *Hp* (DS) for an adult. It resulted that plant consumption by tea infusion was not dangerous for humans, except in the *Hp* for the children with DS. The values of ${\mathrm{CRisk}}_{\mathrm{ccs}}$ were less than 7.57 × 10^−3^—*Hp* (DS), indicating no carcinogenic risk for tea consumption.

The study will be extended to analyze the transfer of elements from food to the human body using the current approach.

## Figures and Tables

**Table 2:** The oral cancer slope factor (SFC) by ingestion exposure (mg/kg/day).

Element	Adult	Product/	References
Cd	6.30	medicinal plant	[78]
Cr	6.10	vegetables	[79]
Cu	0.50	medicinal plant	[46]
Ni	0.84	oral inhalation	[80]

**Table 3:** Mean concentrations of mineral elements in the plants’ inflorescence (mg/kg d.w.) ± standard deviation (SD).

Site	K	Ca	Mg	Na	Fe	Mn	Cu	Zn	Cd
*Sambucus nigra*									
PH	45.52 ± 0.4072	4.47 ± 0.0203	14.61 ± 0.1498	0.01 ± 0.0002	95.91 ± 0.2211	27.17 ± 0.0103	0.41 ± 0.0105	38.60 ± 0.1210	0.001 ± 0.0011
DS	40.14 ± 0.6193	3.89 ± 0.0697	11.44 ± 0.1798	0.07 ± 0.0124	115.40 ± 0.1312	35.74 ± 0.1104	5.49 ± 0.0601	28.22 ± 0.0115	0.08 ± 0.0102
ST	48.67 ± 1.2196	6.85 ± 0.2582	15.82 ± 0.4685	0.02 ± 0.0313	102.70± 0.1501	30.02 ± 0.1642	0.84 ± 0.1005	30.26 ± 0.6404	0.10 ± 0.01121
*Hypericum perforatum*									
PH	0.81 ± 0.0332	2.10 ± 0.0215	0.26 ± 0.0243	0.13 ± 0.0324	113.05 ± 0.1487	133.10 ± 0.1134	11.51 ± 0.1187	26.93 ± 0.0242	0.12 ± 0.0131
DS	0.55 ± 0.0899	1.35 ± 0.0106	0.18 ± 0.0111	0.12 ± 0.0101	193.60 ± 0.2579	107.5 ±0.5143	24.61 ± 0.5096	53.13 ± 0.3232	0.76 ± 0.0242
ST	0.97 ± 0.0389	3.21 ± 0.0313	0.45 ± 0.0231	0.15 ± 0.0211	146.16 ± 0.2032	146.48 ± 0.6010	14.85 ± 0.1789	24.60 ± 0.1115	0.92 ± 0.0223
	*Tilia tomentosa*								
PH	0.84 ± 0.0378	4.83 ± 0.8196	0.17 ± 0.1895	0.145 ± 0.0210	61.39 ± 1.0124	16.67 ± 2.0106	20.56 ± 1.2345	14.91 ± 0.1628	0.08 ± 0.0137
DS	0.63 ± 0.0987	2.56 ± 0.4095	0.12 ± 0.0089	0.086 ± 0.0103	92.83 ± 4.0232	21.32 ± 1.0517	6.07 ± 0.2123	20.56 ± 0.1836	4.67 ± 0.2111
ST	0.98 ± 0.0398	5.78 ± 1.2101	0.26 ± 0.0199	0.104 ± 0.0114	74.62 ± 2.5121	18.42 ± 1.0209	18.62 ± 1.0116	12.46 ± 0.1527	0.12 ± 0.0121

**Table 4:** Maximum transfer rate of mineral elements in the infusion tea (%).

Site	K	Ca	Mg	Na	Fe	Mn	Cu	Zn	Cd
*Sambucus nigra*									
PH	57	15	26	40	12	6	20	30	5
DS	55	25	20	35	15	10	50	50	15
ST	58	36	18	30	10	9	30	25	10
*Hypericum perforatum*									
PH	65	25	36	50	22	70	30	50	15
DS	70	35	40	65	35	90	80	80	35
ST	60	46	28	40	20	77	60	45	20
*Tilia tomentosa*									
PH	50	14	22	32	10	54	18	42	5
DS	52	23	18	30	12	78	66	74	13
ST	54	30	16	26	8	72	54	28	12

**Table 5:** Average chronic daily intake of mineral elements (CDI) (mg/kg of body weight per day).

	*Sambucus nigra*		*Hypericum perforatum*		*Tilia tomentosa*	
Site	Child	Adult	Child	Adult	Child	Adult
PH	4.0 × 10^−3^	1.3 × 10^−3^	9.9 × 10^−3^	2.1 × 10^−3^	1.9 × 10^−3^	0.4 × 10^−3^
DS	4.6 × 10^−3^	1.5 × 10^−3^	1.67 × 10^−2^	3.6 × 10^−3^	3.5 × 10^−3^	0.8 × 10^−3^
ST	4.0 × 10^−3^	1.3 × 10^−3^	1.2 × 10^−2^	2.6 × 10^−3^	2.6 × 10^−3^	0.5 × 10^−3^

**Table 6:** The hazard quotient (HQ) of micro- and trace elements.

	Fe		Mn		Cu		Zn		Cd	
Site	Child	Adult	Child	Adult	Child	Adult	Child	Adult	Child	Adult
*Sambucus nigra*										
PH	1.1 × 10^−2^	3.0 × 10^−3^	8.0 × 10^−3^	2.0 × 10^−3^	1.0 × 10^−3^	0.4 × 10^−3^	2.5 × 10^−3^	8.0 × 10^−3^	0.3 × 10^−4^	0.1 × 10^−4^
DS	1.6 × 10^−2^	5.0 × 10^−3^	1.7 × 10^−2^	5.0 × 10^−3^	4.5 × 10^−2^	1.5 × 10^−2^	3.1 × 10^−3^	1.0 × 10^−2^	8.0 × 10^−3^	3.0 × 10^−3^
ST	1.0 × 10^−2^	3.0 × 10^−3^	1.3 × 10^−2^	4.0 × 10^−3^	4.0 × 10^−3^	1.0 × 10^−3^	1.7 × 10^−3^	5.0 × 10^−3^	7.0 × 10^−3^	2.0 × 10^−3^
*Hypericum perforatum*										
PH	0.6 × 10^−4^	5.0 × 10^−3^	4.4 × 10^−1^	9.4 × 10^−2^	5.6 × 10^−2^	1.2 × 10^−2^	3.0 × 10^−2^	6.3 × 10^−3^	1.2 × 10^−2^	2.5 × 10^−3^
DS	6.4 × 10^−2^	1.4 × 10^−2^	4.6 × 10^−1^	9.8 × 10^−2^	3.2 × 10^−1^	6.9 × 10^−2^	9.3 × 10^−2^	2.0 × 10^−2^	1.7 × 10^−1^	3.75 × 10^−2^
ST	2.7 × 10^−2^	5.9 × 10^−3^	5.3 × 10^−1^	1.1 × 10^−1^	1.5 × 10^−1^	3.1 × 10^−2^	2.4 × 10^−2^	5.2 × 10^−3^	1.2 × 10^−1^	2.6 × 10^−2^
*Tilia tomentosa*										
PH	5.8 × 10^−3^	1.2 × 10^−3^	4.2 × 10^−2^	9.1 × 10^−3^	6.1 × 10^−2^	1.3 × 10^−2^	1.4 × 10^−2^	2.9 × 10^−3^	2.6 × 10^−3^	0.6 × 10^−3^
DS	1.0 × 10^−2^	2.2 × 10^−3^	7.8 × 10^−2^	1.7 × 10^−2^	6.6 × 10^−2^	1.4 × 10^−2^	3.3 × 10^−2^	7.1 × 10^−3^	4.0 × 10^−1^	8.6 × 10^−2^
ST	5.6 × 10^−3^	1.2 × 10^−3^	6.2 × 10^−2^	1.3 × 10^−2^	1.6 × 10^−1^	3.5 × 10^−2^	7.6 × 10^−3^	1.6 × 10^−3^	9.5 × 10^−3^	2.0 × 10^−3^

**Table 7:** The hazard index (HI).

	*Sambucus nigra*		*Hypericum perforatum*		*Tilia tomentosa*	
Site	Child	Adult	Child	Adult	Child	Adult
PH	4.5 × 10^−2^	1.5 × 10^−2^	5.4 × 10^−1^	1.2 × 10^−1^	1.3 × 10^−1^	2.7 × 10^−2^
DS	1.2 × 10^−1^	3.8 × 10^−2^	1.11 × 10^0^	2.4 × 10^−1^	5.9 × 10^−1^	1.3 × 10^−1^
ST	5.0 × 10^−2^	1.6 × 10^−2^	8.49 × 10^−1^	1.8 × 10^−1^	2.5 × 10^−1^	5.4 × 10^−2^

**Table 8:** The cumulative carcinogenic risk of chemical elements (${\mathrm{CRisk}}_{\mathrm{ccs}}$ ).

	*Sambucus nigra*		*Hypericum perforatum*		*Tilia tomentosa*	
Site	Child	Adult	Child	Adult	Child	Adult
PH	2.69 × 10^−5^	8.66 × 10^−6^	1.21 × 10^−3^	2.59 × 10^−4^	1.23 × 10^−3^	2.64 × 10^−4^
DS	9.54 × 10^−4^	3.06 × 10^−4^	7.57 × 10^−3^	1.62 × 10^−3^	3.83 × 10^−3^	8.21 × 10^−4^
ST	1.24 × 10^−4^	4.00 × 10^−5^	3.69 × 10^−3^	7.91 × 10^−4^	3.37 × 10^−3^	7.21 × 10^−4^

**Table 9:** The provisional daily mineral intake (PDMI), mg/kg per day, and RTuiL (%), for adults.

	*Sambucus nigra*		*Hypericum perforatum*		*Tilia tomentosa*	
	PDMI	RTuiL	PDMI	RTuiL	PDMI	RTuiL
PH	3.6 × 10^−3^	0.77	2.10 × 10^−2^	4.52	2.5 × 10^−2^	0.53
DS	4.5 × 10^−3^	0.97	0.76 × 10^−2^	1.63	4.7 × 10^−3^	1.01
ST	2.7 × 10^−3^	0.58	0.27 × 10^−2^	0.58	5.1 × 10^−3^	1.09

## Data Availability

Data will be available on request from the authors.
